# Combination chemotherapy for older patients with unresectable biliary tract cancer: a prospective observational study using propensity-score matched analysis (JON2104-B)

**DOI:** 10.1007/s00535-025-02294-0

**Published:** 2025-09-06

**Authors:** Satoshi Kobayashi, Kohei Nakachi, Kouji Yamamoto, Makoto Ueno, Yuta Maruki, Kenji Ikezawa, Takeshi Terashima, Satoshi Shimizu, Kotoe Oshima, Kunihiro Tsuji, Yoshiharu Masaki, Hidetaka Tsumura, Taro Shibuki, Masato Ozaka, Naohiro Okano, Yukiyasu Okamura, Kumiko Umemoto, Tatsunori Satoh, Yasushi Kojima, Kazuhiko Shioji, Hiroko Nebiki, Toshifumi Doi, Atsushi Naganuma, Shigeki Kataoka, Emiri Kita, Hiroyuki Asama, Kaoru Tsuchiya, Michiaki Unno, Reiko Ashida, Kazuyuki Matsumoto, Izumi Ohno, Takao Itoi, Yuji Negoro, Yasunari Sakamoto, Shiho Arima, Akinori Asagi, Hiroyuki Okuyama, Yoshito Komatsu, Noritoshi Kobayashi, Hiroaki Nagano, Junji Furuse

**Affiliations:** 1 Department of Gastroenterology, Kanagawa Cancer Center, 2-3-2, Nakao, Asahi-ku, Yokohama, 241-0815 Japan; 2 Department of Medical Oncology, Tochigi Cancer Center, Utsunomiya, 320-0834 Japan; 3 Department of Biostatistics, Yokohama City University School of Medicine, Yokohama, 236-0004 Japan; 4 Department of Hepatobiliary and Pancreatic Oncology, National Cancer Center Hospital, Tokyo, 104-0045 Japan; 5 Department of Hepatobiliary and Pancreatic Oncology, Osaka International Cancer Institute, Osaka, 541-8567 Japan; 6 Department of Gastroenterology, Kanazawa University Hospital, Kanazawa, 920-8641 Japan; 7 Department of Gastroenterology, Kanazawa University Hospital, Kita Adaci-Gun, 362-0806 Japan; 8 Division of Gastrointestinal Oncology, Shizuoka Cancer Center, Shunto-gun, 411-8777 Japan; 9 Department of Gastroenterology, Ishikawa Prefectural Central Hospital, Kanazawa, 920-8530 Japan; 10 Department of Gastroenterology and Hepatology, Sapporo Medical University School of Medicine, Sapporo, 060-8556 Japan; 11 Department of Gastroenterological Oncology, Hyogo Cancer Center, Akashi, 673-8558 Japan; 12 Department of Hepatobiliary and Pancreatic Oncology, National Cancer Center Hospital East, Kashiwa, 277-8577 Japan; 13 Hepato-Biliary-Pancreatic Medicine Department, Cancer Institute Hospital of Japanese Foundation for Cancer Research, Tokyo, 135-8550 Japan; 14 Department of Medical Oncology, Kyorin University Faculty of Medicine, Mitaka, 181-8611 Japan; 15 Division of Digestive Surgery, Department of Surgery, Nihon University School of Medicine, Tokyo, 173-8610 Japan; 16 Department of Clinical Oncology, St. Marianna University School of Medicine, Kawasaki, 216-8511 Japan; 17 Department of Gastroenterology, Shizuoka General Hospital, Shizuoka, 420-8527 Japan; 18 Department of Gastroenterology, National Center for Global Health and Medicine, Tokyo, 162-8655 Japan; 19 Department of Gastroenterology, Niigata Cancer Center Hospital, Niigata, 951-8566 Japan; 20 Department of Gastroenterology, Osaka City General Hospital, Osaka, 534-0021 Japan; 21 Department of Molecular Gastroenterology and Hepatology, Kyoto Prefectural University of Medicine, Kyoto, 602-8566 Japan; 22 Department of Gastroenterology, National Hospital Organization Takasaki General Medical Center, Takasaki, 370-0829 Japan; 23 Department of Clinical Oncology, Graduate School of Medicine Faculty of Medicine, Kyoto University, Kyoto, 606-8507 Japan; 24 Department of Gastroenterology, Chiba Cancer Center, Chiba, 260-8717 Japan; 25 Department of Gastroenterology, Fukushima Medical University, Fukushima, 960-1295 Japan; 26 Department of Gastroenterology and Hepatology, Musashino Red Cross Hospital, Musashino, 180-0023 Japan; 27 Department of Surgery, Tohoku University Graduate School of Medicine, Sendai, 980-8574 Japan; 28 Second Department of Internal Medicine, Wakayama Medical University, Wakayama, 641-8509 Japan; 29 Department of Gastroenterology, Okayama University Graduate School of Medicine, Okayama, 700-8558 Japan; 30 Department of Gastroenterology, Chiba University Graduate School of Medicine, Chiba, 260-0856 Japan; 31 Department of Gastroenterology, Tokyo Medical University, Tokyo, 160-0023 Japan; 32 Department of Oncologial Medicine, Kochi Health Sciences Center, Kochi, 781-8555 Japan; 33 Department of Gastroenterology and Hepatology, International University of Health and Welfare Atami Hospital, Atami, 413-0012 Japan; 34 Digestive and Lifestyle Diseases, Kagoshima University Graduate School of Medical and Dental Sciences, Kagoshima, 890-0075 Japan; 35 Department of Gastroenterology, National Hospital Organization Shikoku Cancer Center, Matsuyama, 791-0280 Japan; 36 Department of Medical Oncology, Kagawa University Hospital, Kita-Gun, 761-0793 Japan; 37 Department of Cancer Chemotherapy, Hokkaido University Hospital Cancer Center, Sapporo, 060-8648 Japan; 38 Department of Oncology, School of Medicine Graduate School of Medicine, Yokohama City University, Yokohama, 236-0004 Japan; 39 Department of Gastroenterological, Breast and Endocrine Surgery, Yamaguchi University Graduate School of Medicine, Ube, 755-8505 Japan

**Keywords:** Biliary tract cancer, Unresectable, Chemotherapy, Older, Survival

## Abstract

**Background:**

Systemic chemotherapy with gemcitabine plus S-1 (GEM + S-1), GEM + CDDP plus S-1 (GEM + CDDP + S-1), or gemcitabine plus cisplatin (GEM + CDDP) is standard treatment for advanced biliary tract cancer (aBTC). We aimed to evaluate the efficacy and safety of combination chemotherapy in older patients with aBTC.

**Methods:**

This multicenter prospective observational study (JON2104-B, UMIN000045156) included patients aged ≥ 70 years with aBTC. Inverse-probability weighting propensity-score analyses (IPW) were used to compare overall survival (OS) as the primary endpoint and progression-free survival (PFS) across treatment groups.

**Results:**

This study included 305 patients between August 2021 and January 2023. Of them, 75, 131, 26, 52, and 10 received GEM + CDDP + S-1, GEM + CDDP, GEM + S-1, gemcitabine, and S-1; their median ages were 74, 75, 77.5, 80, and 80 years, and approximately 24%, 16.8%, 23.1%, 9.6%, and 0% had G-8 scores of > 14, respectively. GEM + CDDP had a safety profile comparable to that of GEM + CDDP + S-1 but was more toxic than gemcitabine. Per IPW, the hazard ratio (HR) for GEM + CDDP + S-1 versus GEM + CDDP was 0.80 for OS (95% confidence interval [CI], 0.55–1.17) and 0.55 for PFS (95% CI 0.38–0.80). The HR for GEM + CDDP versus gemcitabine was 0.74 for OS (95% CI 0.42–1.29) and 0.79 for PFS (95% CI 0.42–1.49).

**Conclusions:**

GEM + CDDP + S-1 was associated with longer PFS without additional toxicity than GEM + CDDP for fit older patients. However, the OS for both were not statistically different. The efficacies of GEM + CDDP and gemcitabine for vulnerable older patients did not also differ significantly. These findings highlight the importance of vulnerability in patients with aBTC.

**Supplementary Information:**

The online version contains supplementary material available at 10.1007/s00535-025-02294-0.

## Introduction

Biliary tract cancer (BTC), originating from the biliary epithelium, includes cancers of the gallbladder, intrahepatic bile duct, extrahepatic bile duct, and ampulla of Vater [1]. The incidence of BTC varies worldwide, with BTC being more common in East Asia and Latin America than in North America [2]. In Japan, the fastest aging country in the world, 82% of patients with BTC were ≥ 70 years of age in 2020 [3]. Surgical resection is the only curative treatment for BTC; however, it is not common to be detected at an oncologically resectable stage. Moreover, major hepatectomy, pancreaticoduodenectomy, or both are often required for curative BTC resection. These procedures are too invasive for older patients; therefore, clinical candidates for surgical resection are rare, particularly among older patients with BTC.

Gemcitabine (GEM) has been widely used for systemic chemotherapy for BTC owing to the efficacy and safety with 6–9 months of overall survival (OS) and response rates of 7–18% [4–6]. The ABC-02 trial demonstrated that GEM plus cisplatin (CDDP) had higher OS than GEM monotherapy [7]. BT-22, a Japanese phase II study, reported results similar to those of the ABC-02 [8]. Consequently, GEM + CDDP became the standard treatment for unresectable BTC in 2010. In recent decades, several randomized phase III studies have assessed the efficacy and safety of new treatments as compared to that of GEM + CDDP as reference. OS in JCOG1113 was comparable to that in GEM + CDDP. [9]. Additionally, OS in GEM + CDDP + S-1 was higher than that in GEM + CDDP (hazard ratio (HR) for OS, 0.79; 90% confidence interval (CI), 0.628–0.996) [10]. Recently, immune checkpoint inhibitors have been developed for treatment of unresectable BTC as well as the other types of cancer. Durvalumab or pembrolizumab with GEM + CDDP showed higher OS than GEM + CDDP with HR for OS of 0.80 (95% CI 0.66–0.97) and 0.83 (95% CI 0.72–0.95), respectively.

These pivotal clinical trials were conducted without an upper age limit except JCOG1113 with an upper age limit of 79 years, and the oldest age ranged of 84–85 years; however, the median age in these trials was approximately 65 years, and older patients were a minor population [7, 9–12]. Some studies focused on assessing the efficacy of combination regimens in patients with unresectable BTC. A post hoc analysis of randomized controlled trials (RCTs) revealed that OS in older patients was comparable to that in younger patients [13, 14]. Participants enrolled in RCTs must meet the inclusion criteria, and the older patients included in RCTs may differ from those in the real world. These gaps lead to the need for identifying the optimal regimen for older patients. Therefore, in this study (JON2104-B) we aimed to evaluate the efficacy and safety of combination and monotherapy regimens in older patients with unresectable BTC.

## Methods

### Patients

In this prospective observational study, we enrolled patients from 53 Japanese institutions. The inclusion criteria were: (1) age ≥ 70 years, (2) clinical and pathological diagnosis of BTC including cholangiocarcinoma of intrahepatic, hilar and extrahepatic bile duct, gallbladder carcinoma, and carcinoma of ampulla of Vater, (3) clinical diagnosis of unresectable or recurrent BTC after surgery, (4) scheduled for GEM + CDDP, GEM + S-1, GEM + CDDP + S-1, GEM monotherapy, or S-1 monotherapy, (5) no history of receiving chemotherapy and radiotherapy for BTC; however, patients administered neo-adjuvant and adjuvant chemotherapy for BTC were eligible if the last treatment was administered at least 24 weeks before study initiation, (6) eligible for geriatric assessment, (7) written informed consent obtained for the study, and (8) no concomitant malignant tumor excluding cancers that would not affect the efficacy and safety of treatment for BTC including breast and prostate cancers needing only hormonal treatment. Registration for the study was mandatory before the initiation of systemic chemotherapy for BTCs. This study was approved by the institutional review board of each participating institution. This study was conducted in accordance with the Declaration of Helsinki and the Japanese Ethical Guidelines for Medical and Health Research Involving Human Subjects (clinical trial registration number: UMIN000045156).

### Treatment

Treatment regimens were selected by the attending physician, patient, and their family. Dose modifications and treatment rest were selected at the physician’s discretion during each treatment. The treatments were continued until disease progression, intolerable toxicity, patient refusal, or switching to another adequate therapy including conversion surgery. If first-line treatment was terminated, any subsequent treatment was allowed.

### Assessment of patient characteristics including geriatric assessment

Upon registration, we evaluated the patients’ conditions, including age, height, body weight, sex, clinical stage according to the Union for International Cancer Control Eighth edition, biliary drainage, Eastern Cooperative Oncology Group (ECOG) Performance Status (PS), and laboratory data including neutrophil count, lymphocyte count, and carbohydrate antigen 19–9 (CA 19–9). Moreover, we performed geriatric assessments using the G-8, Instrumental Activity of Daily Living (IADL), and Charlson Comorbidity Index (CCI) to evaluate individual vulnerability beyond chronological age.

### Outcomes of the treatment

The OS, the primary endpoint of this study, was calculated from the date of first-line treatment initiation to the date of death from any cause. Progression-free survival (PFS) was calculated from the date of first-line treatment initiation to the date of documented progression based on radiological or clinical findings or death from any cause. Patients with no progression or death and alive were treated as censored at the time of the last follow-up to evaluate PFS and OS, respectively. Adverse events were evaluated according to the Common Terminology Criteria for Adverse Events, version 5.0. We collected data on the incidence of grade 4 hematological adverse events (AEs) and grade 3–4 non-hematological AEs and the need for emergency hospitalization within three months from first-line treatment initiation. The best radiological response was evaluated using the Response Evaluation Criteria in Solid Tumors version 1.1 [15]. G-8 and IADL were re-evaluated three months after registration as safety endpoints. Relative dose intensity within the first 3 months was calculated as the actual administered dose divided by the planned dose.

### Statistical analysis

We could not estimate how many patients were registered in each treatment group, and the target sample size was set as 300 in total. In case the number of registered patients did not reach the target within one year, the registration period would be extended to a maximum of two years. Data cutoff was planned one year after the last registration. For patient characteristics in each treatment group, continuous variables are expressed as medians with interquartile ranges (IQR), and categorical values are expressed as frequencies with percentages. We performed Fisher’s exact test and the Mann–Whitney *U* test to compare categorical and continuous variables, respectively. We used the Kaplan–Meier method to calculate median OS and PFS with a 95% CI. Log-rank test and Cox regression proportional hazards were used to compare time-to-event parameters between the two groups. The non-deterioration rates of the G-8 and IADL were calculated as the number of patients with equal or higher score at three months than at registration divided by the number of registered patients. To adjust for the patient backgrounds affecting the treatment outcomes, an inverse-probability weighting (IPW) analysis was conducted to compare the efficacy of the different regimens. We used a propensity-score model incorporating age, ECOG PS (0 vs. 1 vs. 2), CA 19–9 level, clinical stage (I/II vs. III vs. IV), primary site (gallbladder vs. others), neutrophil-to-lymphocyte ratio, CRP, and G-8 score to compare GEM + CDDP + S-1 and GEM + CDDP. Age, ECOG PS and CA 19–9 level were used to compare GEM + CDDP and GEM monotherapy. Standardized mean differences and variance ratios were used to evaluate the balance of covariates between the two treatment groups. The groups were considered well balanced when the absolute value of the standardized mean difference was < 0.1 and that of the variance ratio was < 2. All the statistical analyses were performed using SAS, version 9.4 (SAS Institute, Cary, NC, USA). P (p) value less than 0.05 was considered statistically significant.

## Results

### Patients

Overall, a total of 305 patients were enrolled in the study between August 16, 2021, and January 30, 2023. Of these, 11 patients were excluded from the efficacy and safety analysis (8 patients provided the best supportive care although scheduled for chemotherapy, 3 patients did not meet the inclusion criteria). The remaining 294 patients were included in the final analysis (Supplemental Fig. 1).

Initial treatment with GEM + CDDP + S-1, GEM + CDDP, GEM + S-1, GEM monotherapy, and S-1 monotherapy was selected for 75, 131, 26, 52, and 10 patients, respectively. Patient background stratified by treatment group is presented in Table 1. Most of the patients administered combination regimens including GEM + CDDP + S-1, GEM + CDDP, and GEM + S-1 were aged < 80 years, and the median age was around 75 years, while the patients in the monotherapy groups including GEM or S-1 were older than the others and the median age was 80 years and 78 years, respectively. Furthermore, > 70% of the patients had ECOG PS of 0 in GEM + CDDP + S-1 and GEM + CDDP groups; approximately 50% of the patients had ECOG PS of 0 in the other three treatment groups. The proportion of patients categorized as “fit” based on IADL and CCI aligned with that of an ECOG PS of 0 in all the treatment groups. Nonetheless, the proportion of patients with G-8 score > 14 points, which is generally considered “fit older individuals”, was the highest in the GEM + CDDP + S-1 group among all treatment groups; however, only 24.0% of patients in the GEM + CDDP + S-1 group had a G-8 score of > 14 points.

**Table 1:** Patient characteristics

N (%)	Overall	GEM + CDDP + S-1	GEM + CDDP	GEM + S-1	GEM	S-1
	294	75	131	26	52	10
*Age (year)*						
Median [IQR]	76.0 [73.00, 79.00]	74.0 [71.50, 77.00]	75 [73.0, 78.0]	77.5 [73.0, 79.75]	80.0 [76.75, 82.25]	78.0 [76.50, 80.25]
*ECOG PS*						
0	201 (68.4)	57 (76.0)	97 (74.0)	14 (53.8)	28 (53.8)	5 (50.0)
1	81 (27.6)	17 (22.7)	33 (25.2)	9 (34.6)	18 (34.6)	4 (40.0)
2	12 (4.1)	1 (1.3)	1 (0.8)	3 (11.5)	6 (11.5)	1 (10.0)
*Sex*						
Male	189 (64.3)	45 (60.0)	81 (61.8)	20 (76.9)	34 (65.4)	9 (90.0)
*Primary sites*						
Distal bile duct	42 (14.3)	4 (5.3)	21 (16.0)	3 (11.5)	11 (21.2)	3 (30.0)
Intrahepatic bile duct	89 (30.3)	30 (40.0)	36 (27.5)	9 (34.6)	12 (23.1)	2 (20.0)
Hilar bile duct	84 (28.6)	24 (32.0)	40 (30.5)	5 (19.2)	13 (25.0)	2 (20.0)
Gallbladder	70 (23.8)	14 (18.7)	31 (23.7)	9 (34.6)	14 (26.9)	2 (20.0)
Ampulla of Vater	9 (3.1)	3 (4.0)	3 (2.3)	0	2 (3.8)	1 (10.0)
*Disease status*						
Unresectable	232 (78.9)	63 (84.0)	106 (80.9)	20 (76.9)	35 (67.3)	8 (80.0)
Recurrent	62 (21.1)	12(16.0)	25 (19.1)	6 (23.1)	17 (32.7)	2 (20.0)
*Disease stage*						
I	2 (0.7)	1 (1.3)	0 (0.0)	0 (0.0)	1 (1.9)	0 (0.0)
II	19 (6.5)	3 (4.0)	8 (6.1)	3 (11.5)	4 (7.6)	1 (10.0)
III	63 (21.4)	24 (32.1)	27 (20.6)	4 (15.3)	7 (13.4)	1 (10.0)
IV	210 (71.4)	47 (62.7)	96 (73.2)	19 (73.1)	40 (76.9)	8 (80.0)
*Chemotherapy history*						
Present	15 (5.1)	4 (5.3)	6 (4.6)	1 (3.8)	4 (7.7)	0 (0.0)
*Biliary drainage*						
Present	151 (51.4)	39 (52.0)	73 (55.7)	9 (34.6)	23 (44.2)	7 (70.0)
*Pathology*						
Adenocarcinoma	280 (95.2)	73 (97.3)	123 (93.9)	24 (92.3)	51 (98.1)	9 (90.0)
Adenosquamous carcinoma	7 (2.4)	2 (2.7)	2 (1.5)	2 (7.7)	0 (0.0)	1 (10.0)
Others	7 (2.4)	0 (0.0)	6 (4.6)	0 (0.0)	1 (1.9)	0 (0.0)
*CEA (ng/mL)*						
Median [IQR]	3.3 [2.0, 5.9]	3.0 [1.8, 5.5]	3.0 [2.0, 5.3]	3.3 [2.3, 12.9]	3.9 [2.1, 7.0]	5.2 [3.1, 11.3]
*CA 19–9 (U/mL)*						
Median [IQR]	118.9 [23.8, 909.0]	104.0 [35.5, 558.5]	141.0 [28.5, 911.5]	230.0 [23.8, 1303.5]	160.0 [11.2, 1499.5]	19.9 [7.8, 106.2]
*G-8 score*						
Median (point) [IQR]	12.5 [11.0, 14.0]	13.0 [11.0, 14.0]	12.5 [11.0, 14.0]	12.0 [11.0, 13.8]	12.5 [10.0, 14.0]	11.0 [9.3, 12.0]
> 14	51 (17.3)	18 (24.0)	22 (16.8)	6 (23.1)	5 (9.6)	0 (0.0)
*IADL score*						
Full score^†^	217 (73.8)	61 (81.3)	99 (75.6)	19 (73.1)	30 (57.7)	8 (80.0)
*CCI*						
Score = 0	208 (70.7)	56 (74.7)	95 (72.5)	17 (65.4)	36 (69.2)	4 (40.0)

### Treatment efficacy

**Fig. 1:**
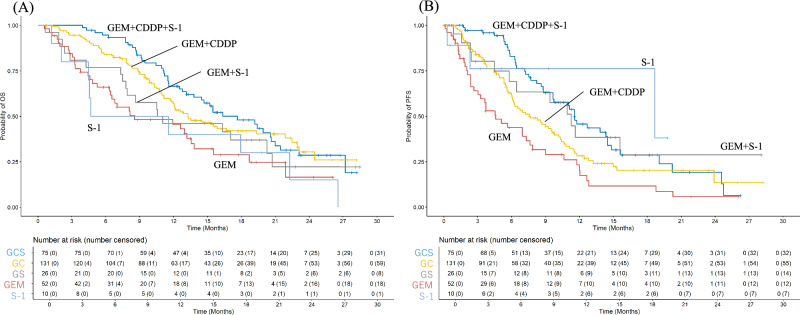
Overall survival (**A**) and progression-free survival (**B**) according to first-line chemotherapy. The blue, yellow, grey, orange, and pale blue lines represent the gemcitabine + cisplatin + S-1, gemcitabine + cisplatin, gemcitabine + S-1, gemcitabine monotherapy, and S-1 monotherapy groups, respectively

As of the data cutoff on 25 January, 2025, median OS was 17.6 months (95% CI 14.5–21.0) for GEM + CDDP + S-1, 13.3 months (95% CI 11.8–21.9) for GEM + CDDP, 10.8 months (95% CI 7.8–not applicable [N.A.]) for GEM + S-1, 8.5 months (95% CI 6.4–15.5) for GEM monotherapy, and 8.1 months (95% CI 4.5–N.A.) for S-1 monotherapy (Fig. 1A). The median PFS was 11.5 (95% CI 9.7–14.8), 7.7 (95% CI 6.1–10.2), 11.3 (95% CI 6.4–N.A.), 4.6 (95% CI 3.0–7.9), and 18.7 months (95% CI 18.7–N.A.) in the GEM + CDDP + S-1, GEM + CDDP, GEM + S-1, GEM monotherapy, and S-1 monotherapy groups, respectively (Fig. 1B). The radiological responses of the treatment groups are presented in Table 2. None of the patients attained CR and PR was observed in 22 (29.3%), 20 (15.3%), 6 (23.1%), 2 (3.8%) and 0 (0%) of the patients treated with GEM + CDDP + S-1, GEM + CDDP, GEM + S-1, GEM monotherapy, and S-1 monotherapy, respectively. SD was observed in 45 (60.0%), 75 (57.3%), 12 (46.2%), 28 (53.8%), and 5 patients (50.0%), and the corresponding disease control rates were 89.3%, 72.6%, 69.3%, 57.6%, and 50.0% in patients treated with GEM + CDDP + S-1, GEM + CDDP, GEM + S-1, GEM monotherapy, and S-1 monotherapy, respectively.

**Table 2:** Radiological response

N (%)	GEM + CDDP + S-1	GEM + CDDP	GEM + S-1	GEM	S-1
CR	0	0	0	0	0
PR	22 (29.3)	20 (15.3)	6 (23.1)	2 (3.8)	0 (0)
SD	45 (60.0)	75 (57.3)	12 (46.2)	28 (53.8)	5 (50.0)
PD	7 (9.3)	24 (18.3)	5 (19.2)	14 (26.9)	4 (40.0)
NE	1 (1.3)	12 (9.2)	3 (11.5)	8 (15.4)	1 (10.0)
Objective response	22 (29.3)	20 (15.3)	6 (23.1)	2 (3.8)	0 (0)
Disease control	67 (89.3)	95 (72.6)	18 (60.3)	30 (57.6)	5 (50.0)

GEM + CDDP + S-1, gemcitabine + cisplatin + S-1, GEM + CDDP, gemcitabine + cisplatin; GEM + S-1, gemcitabine + S-1; GEM, gemcitabine monotherapy; S-1, S-1 monotherapy

### Safety

The AEs reported for the entire cohort are listed in Table 3. The incidence of grade 4 hematological AEs was higher in the three combination treatment groups than that in the two monotherapy groups; moreover, no significant difference in AE incidence was observed among the GEM + CDDP + S-1, GEM + CDDP, and GEM + S-1 groups; however, the GEM monotherapy group had higher incidence of AEs except anemia than that in the S-1 group. The GEM + S-1 group had the highest incidence of grade 3 non-hematological AEs, including malaise and anorexia, followed by the S-1 monotherapy group, and the GEM + CDDP + S-1, GEM + CDDP, and GEM groups had comparable incidence of grade 3 non-hematological AE incidences.

**Table 3:** Adverse events

N (%)	GEM + CDDP + S-1 (*n* = 75)	GEM + CDDP (*n* = 131)	GEM + S-1 (*n* = 26)	GEM (*n* = 52)	S-1 (*n* = 10)
Leukopenia	9 (12.0)	15 (11.5)	3 (11.5)	2 (3.8)	0
Neutrophil count decreased	20 (26.7)	54 (41.2)	8 (30.8)	10 (19.2)	0
Anemia	10 (13.3)	14 (10.7)	2 (7.7)	4 (7.7)	1 (10.0)
Platelet count decreased	8 (10.7)	9 (6.9)	2 (7.7)	1 (1.9)	0
Nausea	1 (1.3)	1 (0.8)	1 (3.8)	1 (1.9)	0
Vomiting	0	0	0	4 (7.7)	1 (10.0)
Anorexia	2 (2.7)	6 (4.6)	2 (7.7)	3 (5.8)	3 (30.0)
Diarrhea	0	0	0	4 (7.7)	1 (10.0)
Fatigue	1 (1.3)	7 (5.3)	2 (7.7)	4 (7.7)	2 (20.0)
Malaise	0	8 (6.1)	4 (15.4)	4 (7.7)	3 (30.0)
Mucositis oral	1 (1.3)	0	0	0	0
Peripheral neuropathy	0	0	0	4 (7.7)	1 (10.0)
Febrile neutropenia	1 (1.3)	1 (0.8)	2 (7.7)	1 (1.9)	0

GEM + CDDP + S-1, gemcitabine + cisplatin + S-1, GEM + CDDP, gemcitabine + cisplatin; GEM + S-1, gemcitabine + S-1; GEM, gemcitabine monotherapy; S-1, S-1 monotherapy

### Treatment course

The data on dose reductions, interruptions, and discontinuations for the treatment groups are presented in Table 4. Initial dose reductions were introduced in 31 (41.3%), 31 (23.7%), 12 (46.2%), 19 (36.5%) and 8 (70.0%) of GEM + CDDP + S-1, GEM + CDDP, GEM + S-1, GEM monotherapy, and S-1 monotherapy groups, respectively. Dose interruptions were needed in 41 (54.7%), 98 (74.8%), 22 (84.6%), 40 (76.9%), and 5 patients (50.0%) in the GEM + CDDP + S-1, GEM + CDDP, GEM + S-1, GEM monotherapy, and S-1 monotherapy groups, respectively. Dose reductions after initiation were needed in 26 (34.7%), 48 (36.6%), 9 (34.6%), 8 (15.4%), and 0 patients (0%) in the GEM + CDDP + S-1, GEM + CDDP, GEM + S-1, GEM monotherapy, and S-1 monotherapy groups, respectively. Unexpected hospitalization due to AEs was required in 9 (12.0%), 33 (25.2%), 6 (23.1%), 13 (25.0%), and 2 patients (30.0%) in the GEM + CDDP + S-1, GEM + CDDP, GEM + S-1, GEM monotherapy, and S-1 monotherapy groups, respectively. At the data cutoff, first-line treatment was discontinued in 64 (85.3%), 117 (89.3%), 22 (84.6%), 49 (94.2%), and 9 (90.0%) patients in the GEM + CDDP + S-1, GEM + CDDP, GEM + S-1, GEM monotherapy, and S-1 monotherapy groups. The most common reason for discontinuation was disease progression, followed by AEs. Other reasons included switching treatment to GEM + CDDP with immune checkpoint inhibitors. Treatment discontinuation due to AEs was observed more frequently in patients treated with regimens including S-1. Subsequent treatment was introduced in 27 (36.0%), 61 (46.6%), 12 (46.2%), 21 (40.4%), and 1 (10.0%) patients in the GEM + CDDP + S-1, GEM + CDDP, GEM + S-1, GEM monotherapy, and S-1 monotherapy groups, respectively.

**Table 4:** Treatment course and reason for treatment discontinuation

N (%)	GEM + CDDP + S-1 (*n* = 75)			GEM + CDDP (*n* = 131)		GEM + S-1 (*n* = 26)		GEM (*n* = 52)	S-1 (*n* = 10)
Initial dose reduction	31 (41.3)			31 (23.7)		12 (46.2)		19 (36.5)	8 (70.0)
Dose interruption	41 (54.7)			98 (74.8)		22 (84.6)		40 (76.9)	5 (50.0)
Dose reduction after initiation	26 (34.7)			48 (36.6)		9 (34.6)		8 (15.4)	0 (0)
Relative dose intensity, mean (%)^†^	GEM	CDDP	S-1	GEM	CDDP	GEM	S-1	GEM	S-1
	92.7	89.4	87.2	80.4	77.3	82.2	66.7	78.7	72.8
Unexpected hospitalization‡	9 (12.0)			33 (25.2)		6 (23.1)		13 (25.0)	3 (30.0)
Discontinued at data cutoff	64 (85)			117 (89)		22 (85)		49 (94)	9 (90)
Reason for discontinuation									
Disease progression	44 (69)			76 (65)		12 (55)		39 (80)	3 (33)
Adverse events	14 (22)			20 (17)		8 (36)		6 (12)	6 (67)
Conversion surgery	3 (5)			4 (3)		1 (5)		1 (2)	0
Others	3 (5)			17 (15)		1 (5)		3 (6)	0

^†^Within the first three months

^‡^Hospitalization that physicians considered to be treatment-related adverse events

### Changes in G-8 and IADL scores

Geriatric assessment was evaluable after 3 months in 70 (93.3%), 116 (88.5%), 19 (73.1%), 40 (76.9%), and 7 (70.0%) patients in the GEM + CDDP + S-1, GEM + CDDP, GEM + S-1, GEM monotherapy, and S-1 monotherapy groups, respectively. The non-deterioration rates of G-8 and IADL were higher in the GEM + CDDP + S-1 group than that in the GEM + CDDP and GEM + S-1 groups, and they were the lowest in the S-1 group among all the treatment groups. The non-deterioration rates of G-8 were 58.7%, 55.7%, 34.6%, 42.3% and 20.0% and that of IADL were 82.7%, 73.3%, 42.3%, 57.7% and 40.0%, in the GEM + CDDP + S-1, GEM + CDDP, GEM + S-1, GEM monotherapy, and S-1 monotherapy groups, respectively.

### Comparison between GEM + CDDP + S-1 and GEM + CDDP using propensity-score matched analysis

**Fig. 2:**
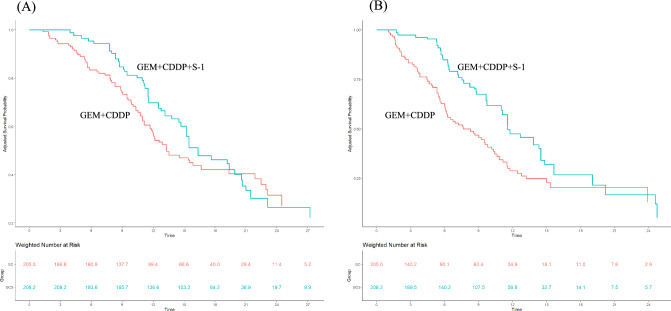
Comparison of overall survival (**A**) and progression-free survival (**B**) between gemcitabine + cisplatin + S-1 and gemcitabine + cisplatin groups using propensity-score-matched analysis. The blue and orange line represents gemcitabine + cisplatin + S-1 and gemcitabine + cisplatin groups, respectively

IPW adjustment revealed that all absolute mean differences between the GEM + CDDP + S-1 and GEM + CDDP groups were < 0.1, indicating that patients of these two regimens were well balanced (Supplemental Fig. 2). The variance ratio of the propensity-score between the treatment groups showed improved covariate balance after IPW adjustment (Supplemental Table 1). The median OS was 16.4 (95% CI 14.5–20.6) months in GEM + CDDP + S-1 group and 13.3 (95% CI 11.8–21.9) months in GEM + CDDP group (Fig. 2A). The HR of OS in the GEM + CDDP + S-1 group as compared to that of GEM + CDDP group was 0.81 (95% CI 0.56–1.18; *p* = 0.282); additionally, the median PFS was 11.6 (95% CI 9.7–14.5) and 8.3 (95% CI 6.0–10.2) months in the GEM + CDDP + S-1 and GEM + CDDP groups, respectively (Fig. 2B). The HR of PFS was 0.56 (95% CI 0.39–0.81; *p* = 0.002). Objective response was observed in 31.0% and 15.5% of the patients in the GEM + CDDP + S-1 and GEM + CDDP groups, respectively with statistically significant difference in objective response rate between the two groups (95% CI 9–22; *p* < 0.001).

### Comparison between GEM + CDDP and GEM with propensity-score matched analysis

**Fig. 3:**
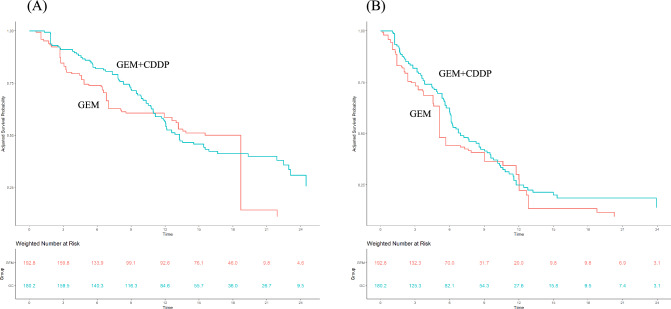
Comparison of overall survival (**A**) and progression-free survival (**B**) between gemcitabine + cisplatin and gemcitabine monotherapy groups using propensity-score-matched analysis. The blue and orange line represents gemcitabine + cisplatin and gemcitabine monotherapy groups, respectively

After IPW adjustment, all absolute mean differences were < 0.1, indicating that patients in the GEM + CDDP and GEM groups were well balanced (Supplemental Fig. 3). The variance ratio of the propensity scores between the treatment groups showed improved covariate balance after IPW adjustment (Supplementary Table 2). The median OS was 13.3 (95% CI 11.1–19.4) and 15.5 (95% CI 6.4–18.7) months in the GEM + CDDP and GEM groups, respectively (Fig. 3A). The HR of OS in the GEM + CDDP group as compared to that in the GEM group was 0.74 (95% CI 0.42–1.29; *p* = 0.282); additionally, the median PFS was 6.9 (95% CI 6.0–9.6) and 5.1 (95% CI 3.0–12.0) months in the GEM + CDDP and GEM group, respectively (Fig. 3B). The HR of PFS was 0.79 (95% CI 0.42–1.49; *p* = 0.463). Objective responses were observed in 14.1% and 7.5% of the patients in the GEM + CDDP and GEM group, respectively; however, the difference in objective response rate was not statistically significant (95% CI -6–19; *p* = 0.305).

## Discussion

In this multicenter, prospective, observational study, we characterized the safety and efficacy of systemic chemotherapy in older patients with aBTC. The combination regimens including GEM + CDDP + S-1, GEM + CDDP and GEM + S-1 were administered mainly in patients aged ≤ 80 years, and patients aged > 80 years preferred monotherapy including GEM and S-1. Regarding the efficacy of GEM + CDDP + S-1 as compared to that of GEM + CDDP, no statistically significant difference was observed in OS; however, statistically significant difference was observed between the two treatment groups in PFS. Although the initial reduction was applied more frequently with GEM + CDDP + S-1 group than that in the GEM + CDDP group, the safety of the two treatment regimens seemed comparable. The efficacies of GEM + CDDP and GEM were not different in terms of OS, PFS, and response rate; however, GEM + CDDP were associated with severe AEs, requiring treatment modification, and increased vulnerability. Based on these results, we suggest that GEM + CDDP + S-1 should be considered the standard treatment even for fit patients aged > 70 years, while GEM could serve as an alternative to GEM + CDDP for vulnerable older patients.

Regarding the items reported in general clinical trials, including age, sex, ECOG PS, and CA 19–9 level, the patient characteristics were comparable in the GEM + CDDP + S-1 and GEM + CDDP groups in our study; however, the proportion of patients with G-8 > 14 points, full IADL score, and CCI = 0 points were consistently higher in the GEM + CDDP + S-1 group than that in the GEM + CDDP group, indicating that GEM + CDDP + S-1 was more frequently selected in fit older patients. Therefore, we incorporated G-8 score as a covariate in the propensity-score model. Moreover, > 70% of the patients in GEM + CDDP + S-1 and GEM + CDDP groups had an ECOG PS of 0; while, approximately 20% of patients had G-8 > 14 points. The results indicated that many patients were suspected of being vulnerable even with an ECOG PS of 0. These results indicated the importance of geriatric assessment in older patients to identify vulnerabilities, which cannot be captured by routine oncology assessments. Subgroup analyses stratified by G-8 and IADL would validate the importance of vulnerability defined by such geriatric assessments, and we plan to perform these analyses in future.

Although 40% of patients in the GEM + CDDP + S-1 group demonstrated an initial dose reduction, there were fewer treatment interruptions in this group than in the GC group and the RDI was maintained. We speculate that this was a reason why OS, PFS, and ORR were clinically and/or significantly better in the GEM + CDDP + S-1 group than in the GEM + CDDP group, with comparable efficacy and better safety compared to that in the KHBO-1401 phase III trial. Furthermore, the incidence of severe toxicities was not frequent in the GEM + CDDP + S-1 group as compared to that in the GEM + CDDP group. This improved efficacy and safety may have resulted in a higher non-deterioration rate of G-8 and IADL for GEM + CDDP + S-1 than that for GEM + CDDP. Based on these results, we believe that GEM + CDDP + S-1 can be the standard treatment for patients aged > 70 years as well as for younger patients [10]. Nonetheless, the cause of more frequent initial dose reduction and treatment discontinuation due to AEs observed with GEM + CDDP + S-1 than that with GEM + CDDP, needs to be characterized. Moreover, personalized medicine is preferred in older patients owing to the extensive differences among individuals, even within the same age group [16, 17]. Therefore, we plan future subgroup analysis to evaluate the effect of individual background characteristics and initial dose reductions on efficacy and safety.

During comparison of the efficacy between GEM + CDDP and GEM, primary cancer sites were not incorporated into the model of the IPW as the patient background was biased and the model did not fit well on incorporation of primary sites (Supplemental Fig. 4). No significant differences were observed in the OS, PFS, or ORR between GEM + CDDP and GEM, which were numerically comparable. In the GEM + CDDP group, the incidence of severe AEs and treatment discontinuation due to toxicities was higher, and the non-deterioration rate of geriatric assessment scores was lower than that in the GEM group despite the better patient background in the GEM + CDDP group. These results consistently indicated that GEM + CDDP was more toxic than GEM in older patients. Based on the balance between efficacy and safety, GEM seemed to be more favorable than GEM + CDDP in older patients involved in our study. McNamara M.G. et al. reported that combination therapy was associated with better OS and PFS than monotherapy in older patients aged ≥ 70 years [13]. The difference in the results between their study and ours might be due to the differences in the patient backgrounds. Their study was a post hoc analysis of RCTs that compared combination therapy and monotherapy, and only specific older patients might have been enrolled in such trials based on the eligibility criteria despite detailed patient characteristics being unavailable in their study [13]. In contrast, most of patients in the GEM group in our study seemed vulnerable; the median age was 80 years, the proportion of the patients with ECOG PS of 0 was approximately half, and < 10% of the patients had G-8 score > 14 points. Therefore, we suggest that GEM can be an alternative treatment for GEM + CDDP in older vulnerable patients aged ≥ 70 years.

In our study, few patients were administered GEM + S-1 and S-1 monotherapy. Although the efficacy of S-1 monotherapy was not compared with that of GEM monotherapy using a propensity-score-matched analysis, OS associated with these two monotherapies seemed comparable. Regarding the GEM + S-1 group, the PFS, ORR, and DCR were similar to those of the GEM + CDDP group. Considering differences in patient backgrounds including older age and poorer PS in the GEM + S-1 group than in the GEM + CDDP group, the OS for GEM + S-1 would not be inferior to that for GEM + CDDP. The efficacy of S-1 monotherapy and GEM + S-1 has been reported to be comparable to that of GEM monotherapy and GEM + CDDP, respectively [7–9] in cohorts where most enrolled patients were < 70 years of age; nevertheless, these data support our results that S-1 monotherapy and GEM + S-1 could be alternatives for GEM monotherapy and GEM + CDDP, respectively, in older patients. However, S-1 toxicity has been reported in patients with renal dysfunction, including those with creatinine clearance of < 50 mL/min [18, 19], and deteriorated renal function is reported more frequently in older adults. Therefore, S-1 administration should be considered carefully in older patients. Moreover, initial dose reduction was more frequently adopted in patients administered S-1 containing regimens (GEM + CDDP + S-1, GEM + S-1 and S-1) than in those administered other regimens. Furthermore, treatment discontinuation due to AEs was more frequent in patients administered S-1 containing regimens than those administered other regimens despite the initial dose reduction, as the AE profile in S-1 was mainly gastrointestinal toxicity, including nausea, anorexia, and diarrhea. Therefore, careful monitoring and treatment adjustment based on AE is important in older patients receiving S-1 containing regimens.

Our study had some limitations. First, this was an observational study; therefore, the patients’ backgrounds were different in each treatment group, and dose reduction and interruption were not controlled. These issues can be resolved using RCT. However, conducting RCT with older patients is difficult owing to variations in their willingness to undergo treatment, and the limited capacity of some patients to provide informed consent. Thus, patients with poor general condition and comorbidities, important in the real world will not be enrolled in RCTs [20]. Therefore, a prospective observational design was selected for this study. Furthermore, we believe that comparability between different regimens was ensured because the many institutions participated in this study and the IPW method was used to adjust for potential differences in patient backgrounds although potential prognostic factors, such as the presence of biliary drainage, were still uncontrolled. Second, the number of patients, particularly in the GEM + S-1 and S-1 monotherapy groups, was small for conducting direct comparisons with other treatment groups. Third, we did not include patients administered GEM + CDDP with an immune checkpoint inhibitor (ICI) as a first-line treatment. In recent years, both TOPAZ-1 and KENOTE-966 studies have demonstrated the superiority of OS in GEM + CDDP + ICI over GEM + CDDP [11, 12], and these have become standard treatments worldwide. However, the patient enrollment in our study was completed before ICIs were introduced into clinical practice. We believe that the results of our study are still useful in this ICI era owing to the contraindication of ICIs in some older patients based on poor adherence to ICI treatment and their comorbidities, including autoimmune disorders. Furthermore, the AE profile associated with ICI rarely overlaps with that associated with GEM + CDDP; our study results therefore provide insights into the potential of GEM + CDDP + ICI in older patients. The efficacy and safety of GEM + CDDP + ICI and GEM + CDDP + S-1 still need to be compared in older and younger patients, and a randomized controlled study comparing the two triplet treatments is ongoing (jRCT1031240320). Fourth, we evaluated only grade 3 or higher AEs, making it unclear how lower-grade (≤ grade 2) AEs may have affected treatment.

In conclusion, treatment selection in the clinical practice of older patients varied widely based on each patient’s background, including geriatric assessment scores. GEM + CDDP + S-1 was associated with a marginal benefit in OS and a statistically significant improvement in PFS as compared to that associated with GEM + CDDP in fit older patients aged > 70 years, and GEM may be an alternative treatment to GEM + CDDP based on the balance of efficacy and safety in vulnerable older patients.

## Supplementary Information

Below is the link to the electronic supplementary material.

Fig. S1 Patient recruitment flow

Fig. S2 Standardized mean differences between the gemcitabine+cisplatin+S-1 and gemcitabine+cisplatin groups after inverse-probability-weighted adjustment

Fig. S3 Standardized mean differences between gemcitabine+cisplatin and gemcitabine monotherapy after inverse-probability-weighted adjustment

Fig. S4 Standardized mean differences between GEM+CDDP and GEM monotherapy after inverse-probability-weighted adjustment using the model including the primary tumor sites. Patient backgrounds were not well balanced on inclusion of age, ECOG PS, CA 19-9 level and primary sites of the tumor in the model.

Supplementary file 5 (DOCX 20 KB)

Supplementary file 6 (DOCX 19 KB)
