# Supplementary material for: Combination chemotherapy for older patients with unresectable biliary tract cancer: a prospective observational study using propensity-score matched analysis (JON2104-B)
Source: J Gastroenterol. 2025 Sep 6;60(12):1584–95. doi: 10.1007/s00535-025-02294-0 (PMC12630146; doi:10.1007/s00535-025-02294-0)
Supplement: Supplementary file 1 — Fig. S1 Patient recruitment flow [file 535_2025_2294_MOESM1_ESM.pptx]

## Slide 1
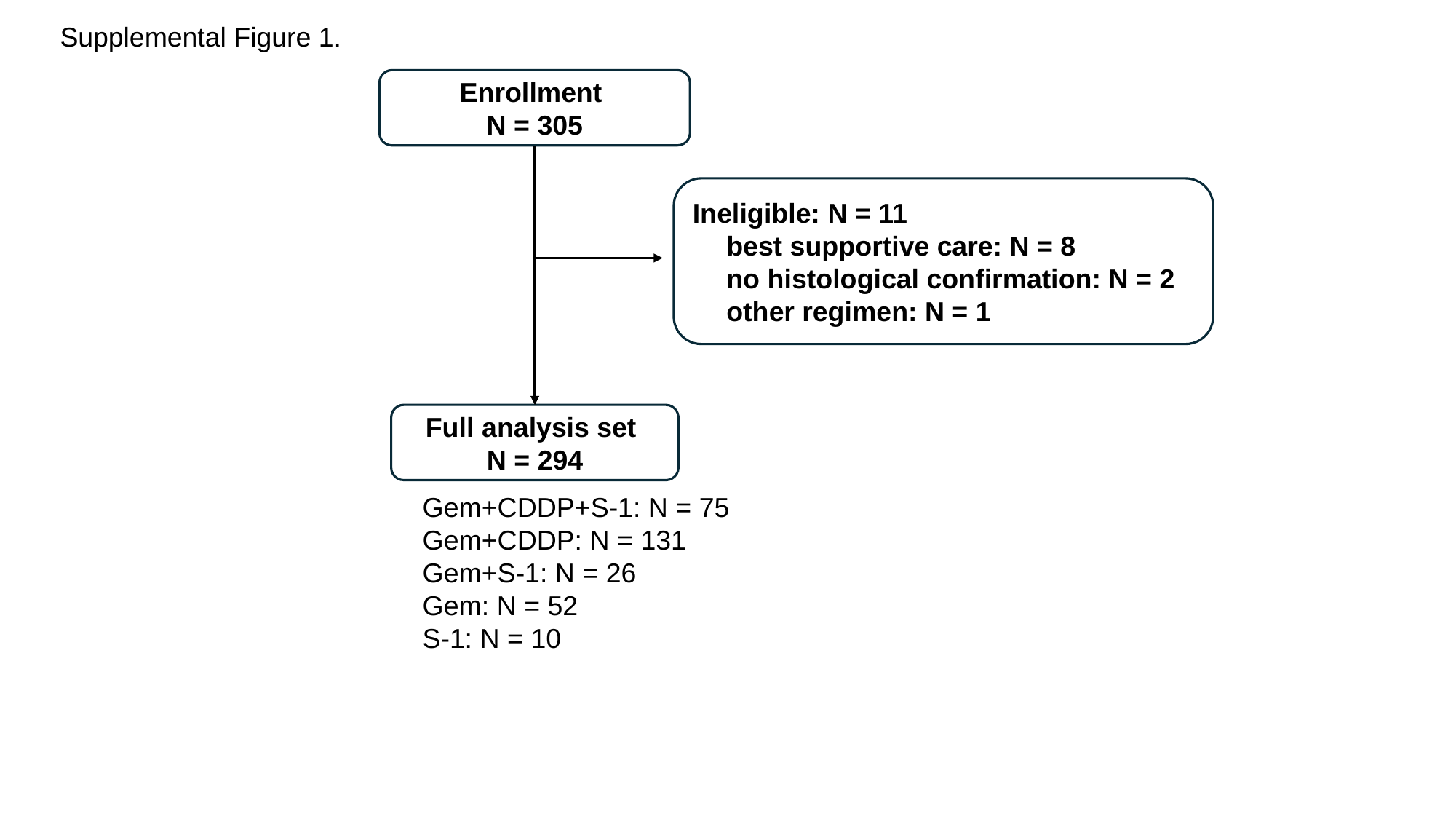

Supplemental Figure 1.
Enrollment
N = 305
Ineligible: N = 11
　best supportive care: N = 8
　no histological confirmation: N = 2
　other regimen: N = 1
Full analysis set
N = 294
Gem+CDDP+S-1: N = 75
Gem+CDDP: N = 131
Gem+S-1: N = 26
Gem: N = 52
S-1: N = 10
