# Supplementary material for: Combination chemotherapy for older patients with unresectable biliary tract cancer: a prospective observational study using propensity-score matched analysis (JON2104-B)
Source: J Gastroenterol. 2025 Sep 6;60(12):1584–95. doi: 10.1007/s00535-025-02294-0 (PMC12630146; doi:10.1007/s00535-025-02294-0)
Supplement: Supplementary file 2 — Fig. S2 Standardized mean differences between the gemcitabine+cisplatin+S-1 and gemcitabine+cisplatin groups after inverse-probability-weighted adjustment [file 535_2025_2294_MOESM2_ESM.pptx]

## Slide 1
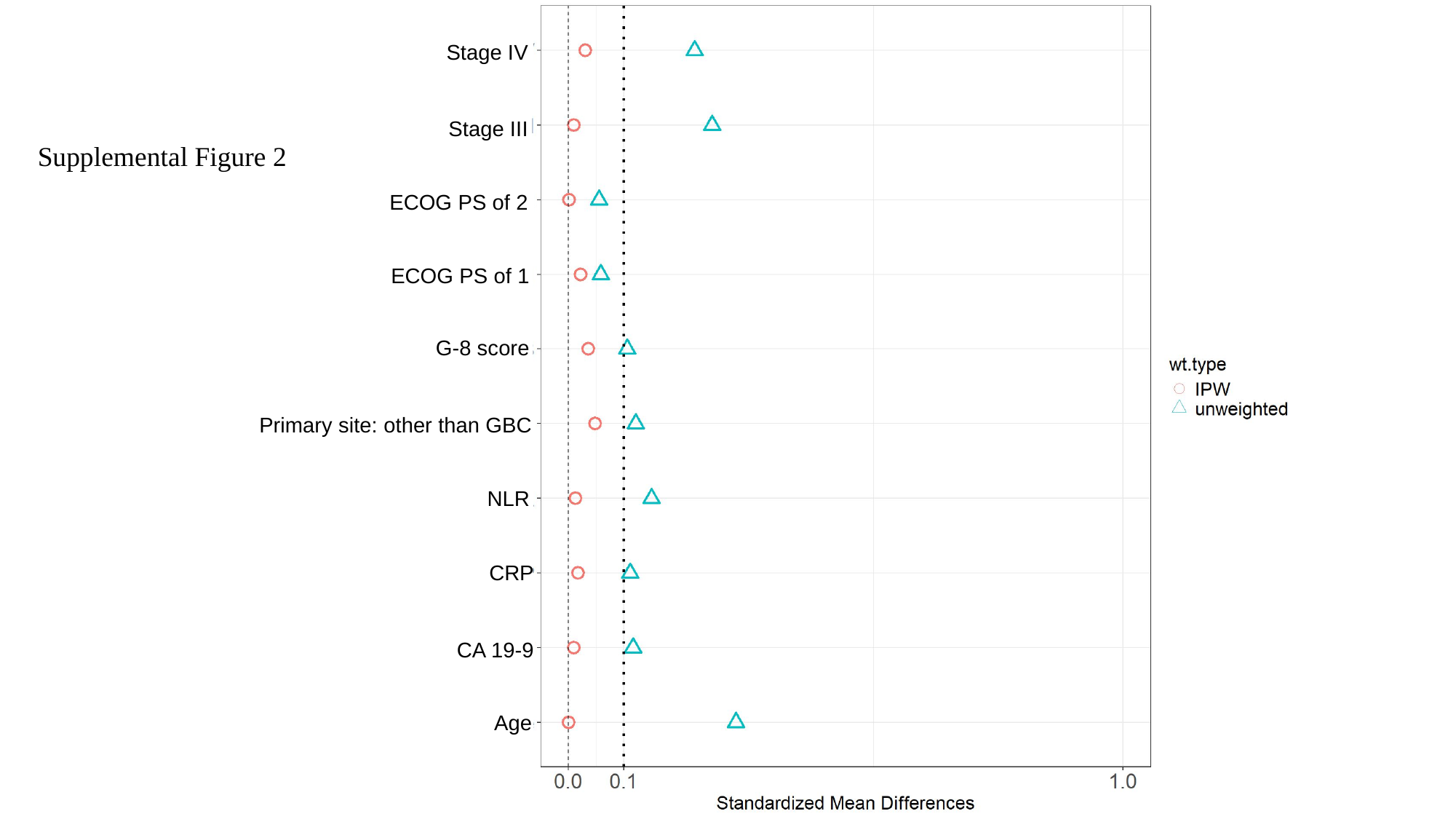

Stage IV
#
Stage III
Supplemental Figure 2
ECOG PS of 2
ECOG PS of 1
G-8 score
Primary site: other than GBC
NLR
CRP
CA 19-9
Age
