# Supplementary figures and images for: Combination chemotherapy for older patients with unresectable biliary tract cancer: a prospective observational study using propensity-score matched analysis (JON2104-B)
Source: J Gastroenterol. 2025 Sep 6;60(12):1584–95. doi: 10.1007/s00535-025-02294-0 (PMC12630146; doi:10.1007/s00535-025-02294-0)

## Slide 1
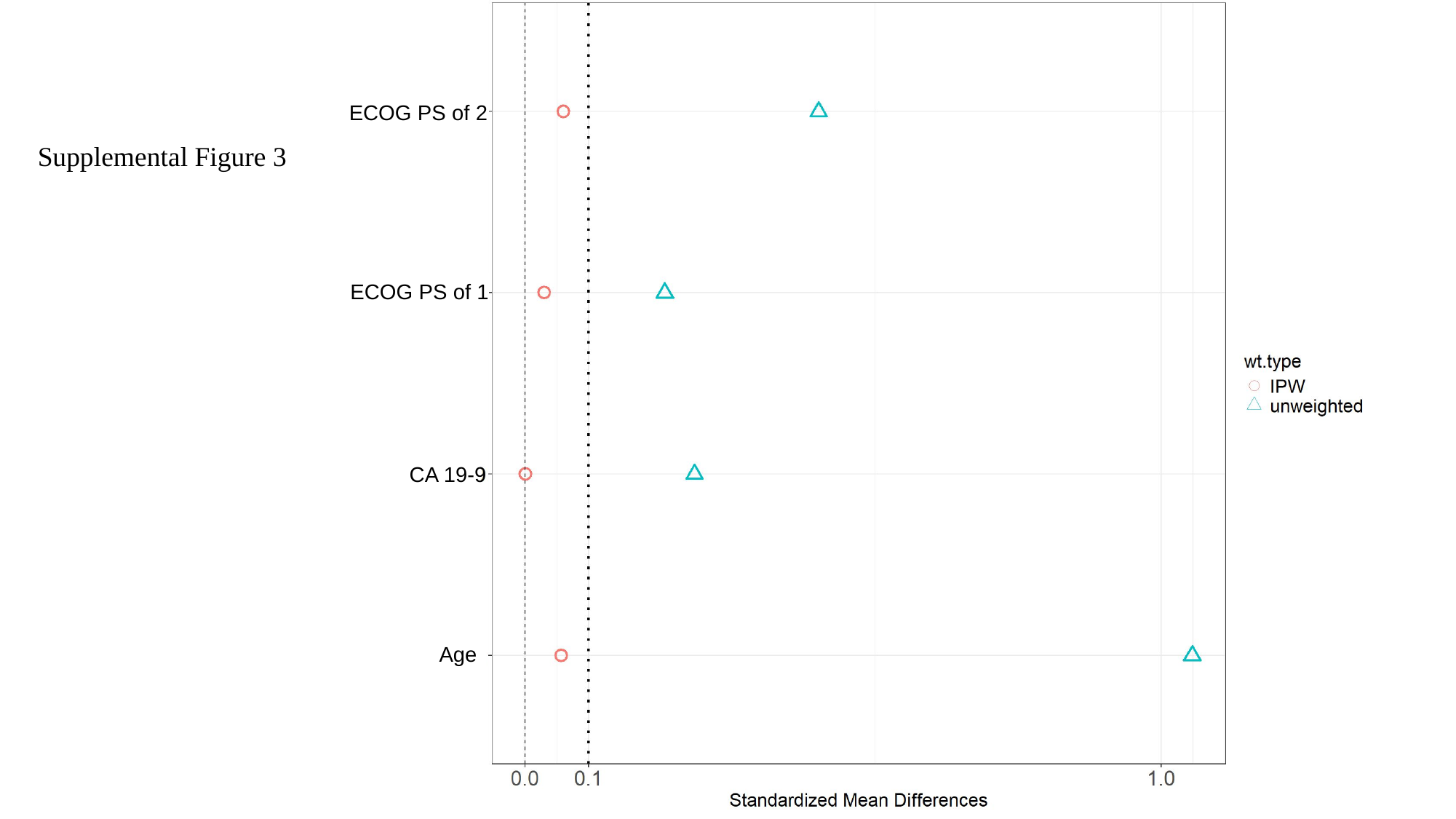

ECOG PS of 2
Supplemental Figure 3
ECOG PS of 1
CA 19-9
Age

Supplement: Supplementary file 3 — Fig. S3 Standardized mean differences between gemcitabine+cisplatin and gemcitabine monotherapy after inverse-probability-weighted adjustment [file 535_2025_2294_MOESM3_ESM.pptx]

## Slide 1
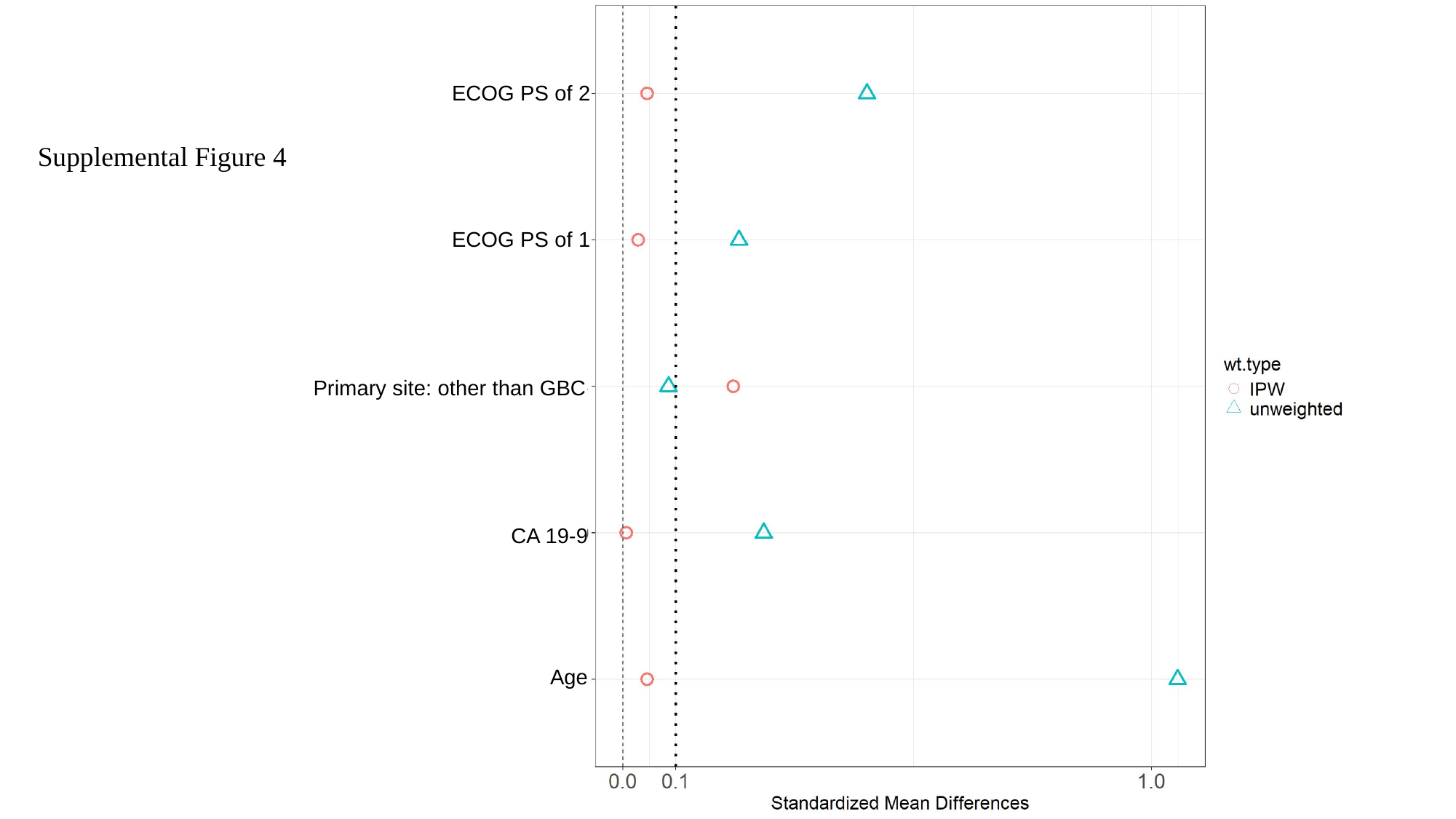

ECOG PS of 2
Supplemental Figure 4
ECOG PS of 1
Primary site: other than GBC
CA 19-9
Age

Supplement: Supplementary file 4 — Fig. S4 Standardized mean differences between GEM+CDDP and GEM monotherapy after inverse-probability-weighted adjustment using the model including the primary tumor sites. Patient backgrounds were not well balanced on inclusion of age, ECOG PS, CA 19-9 level and primary sites of the tumor in the model. [file 535_2025_2294_MOESM4_ESM.pptx]
