# Supplementary material for: Combination chemotherapy for older patients with unresectable biliary tract cancer: a prospective observational study using propensity-score matched analysis (JON2104-B)
Source: J Gastroenterol. 2025 Sep 6;60(12):1584–95. doi: 10.1007/s00535-025-02294-0 (PMC12630146; doi:10.1007/s00535-025-02294-0)
Supplement: Supplementary file 5 — (DOCX 20 KB) [file 535_2025_2294_MOESM5_ESM.docx]

Supplemental Table 1. The variance ratio of the propensity-score between the GEM+CDDP+S-1 and GEM+CDDP groups

|  | GEM+CDDP+S-1 | GEM+CDDP | SMD |
| --- | --- | --- | --- |
| Age (mean [SD]) | 75.17 (3.44) | 75.17 (3.55) | 0.001 |
| ECOG PS (%)  0  1  2 | 73.6  25.4  0.9 | 74.6  24.4  0.9 | 0.023 |
| Primary sites (%)  Gallbladder  The others | 19.6  80.4 | 21.6  78.4 | 0.049 |
| CA 19-9 (mean [SD]) | 2863.05 (19372.00) | 2703.13 (8452.88) | 0.011 |
| Disease stage (%)  I/II  III  IV | 8.1  23.7  68.2 | 6.2  24.1  69.7 | 0.073 |
| NLR (mean [SD]) | 3.28 (2.04) | 3.31 (2.31) | 0.013 |
| CRP (mean [SD]) | 1.20 (2.39) | 1.24 (2.09) | 0.018 |
| G-8 score (mean [SD]) | 12.37 (2.38) | 12.45 (2.26) | 0.036 |

SD, standard deviation; ECOG PS, Eastern Cooperative Oncology Group performance status; CA 19-9, carbohydrate antigen 19-9; NLR, neutrophil-lymphocyte ratio; CRP, C-reactive protein; GEM+CDDP+S-1, gemcitabine+cisplatin+S-1; GEM+CDDP, gemcitabine+cisplatin; SMD, standardized mean difference
