# Supplementary material for: Combination chemotherapy for older patients with unresectable biliary tract cancer: a prospective observational study using propensity-score matched analysis (JON2104-B)
Source: J Gastroenterol. 2025 Sep 6;60(12):1584–95. doi: 10.1007/s00535-025-02294-0 (PMC12630146; doi:10.1007/s00535-025-02294-0)
Supplement: Supplementary file 6 — (DOCX 19 KB) [file 535_2025_2294_MOESM6_ESM.docx]

Supplemental Table 2. The variance ratio of the propensity scores between the GEM+CDDP and GEM groups

|  | GEM+CDDP | GEM | SMD |
| --- | --- | --- | --- |
| Age (mean [SD]) | 76.47 (3.87) | 76.23 (4.42) | 0.058 |
| ECOG PS (%)  0  1  2 | 66.6  28.4  4.9 | 69.2  27.1  3.7 | 0.073 |
| CA 19-9 (mean [SD]) | 2977.72 (7711.94) | 2970.93 (16465.16) | 0.001 |

SD, standard deviation; ECOG PS, Eastern Cooperative Oncology Group performance status; CA 19-9, carbohydrate antigen 19-9; SMD, standardized mean difference
